# Insight into the Molecular Mechanism of the Transcriptional Regulation of *amtB* Operon in *Streptomyces coelicolor*

**DOI:** 10.3389/fmicb.2018.00264

**Published:** 2018-02-20

**Authors:** Zhendong Li, Xinqiang Liu, Jingzhi Wang, Ying Wang, Guosong Zheng, Yinhua Lu, Guoping Zhao, Jin Wang

**Affiliations:** ^1^CAS Key Laboratory of Synthetic Biology, Institute of Plant Physiology and Ecology, Shanghai Institutes for Biological Sciences, Chinese Academy of Sciences, Shanghai, China; ^2^University of Chinese Academy of Sciences, Beijing, China; ^3^State Key Laboratory of Genetic Engineering, Department of Microbiology and Microbial Engineering, School of Life Sciences, Fudan University, Shanghai, China; ^4^Department of Microbiology, Prince of Wales Hospital, The Chinese University of Hong Kong, Shatin, Hong Kong; ^5^Li Ka Shing Institute of Health Sciences, Prince of Wales Hospital, The Chinese University of Hong Kong, Shatin, Hong Kong

**Keywords:** *Streptomyces coelicolor*, GlnR box, *amtB*, transcriptional regulation, nitrogen metabolism, GlnR

## Abstract

In *Streptomyces coelicolor*, *amtB* transcription is promptly regulated by the global nitrogen regulator GlnR. Although the GlnR binding *cis*-element has been characterized in *amtB* promoter, consisting of three GlnR boxes of *a3-b3*, *a1-b1*, and *a2-b2*, its role in GlnR-mediated transcriptional regulation remains unclear. Here, we showed that GlnR had different binding affinity against each pair of GlnR binding sites in *amtB* promoter (i.e., *a3-b3*, *a1-b1*, and *a2-b2* sites), and GlnR was able to bind *a3-b3* and *a1-b1*, respectively, but not *a2-b2* alone. Consistently, *a2* was not a typical GlnR binding site and further experiments showed that *a2* was non-essential for GlnR-mediated binding *in vitro* and transcriptional regulation *in vivo*. To uncover the physiological role of the three GlnR boxes, we then mutated the wild-type *amtB* promoter to a typical GlnR-binding motif containing two GlnR boxes (*a3-b3–a2-b2*), and found although the transcription of the mutated promoter could still be activated by GlnR, its increasing rate was less than that of the wild-type. Based on these findings, one could conclude that the three GlnR boxes assisted GlnR in more promptly activating *amtB* transcription in response to nitrogen limitation, facilitating bacterial growth under nitrogen stresses.

## Introduction

Soil-dwelling actinomycetes produce a large number of bioactive secondary metabolites, including antibiotics, immunosuppressants, and antitumor agents (Berdy, 2005). Biosynthesis of these metabolites has been found to be regulated by the availability of carbon, nitrogen, and phosphate sources (Martin and Demain, 1980; Hodgson, 2000), among which the concentration of utilizable nitrogen is particularly pivotal (Reuther and Wohlleben, 2007).

Nitrogen is an essential element for bacterial growth and an important component of cellular biological macromolecules such as amino acids, nucleotides, and cell wall components (Merrick and Edwards, 1995). Consequently, bacteria have developed complex transcriptional regulatory systems to allow them to sense internal and external nitrogen levels, coordinating the expression of genes involved in nitrogen metabolism and helping bacteria adapt to environmental stress (Leigh and Dodsworth, 2007). In enteric bacteria, the regulation of nitrogen metabolism is mediated by the NtrB–NtrC two-component system (Reitzer, 2003). However, in *Streptomyces coelicolor*, a model actinomycete, the global nitrogen metabolism is stringently regulated by the OmpR-type regulator, GlnR (Fink et al., 2002; Tiffert et al., 2008, 2011). Under nitrogen-limited conditions, GlnR activates the transcription of its targets and thus promotes nitrogen metabolisms (Tiffert et al., 2008), which may help bacteria cope with the nitrogen deficiency.

As an orphan response regulator, GlnR has no cognate kinase and is post-translationally modified by phosphorylation and acetylation (Wray and Fisher, 1993; Lin et al., 2014; Amin et al., 2016). GlnR homologs are widely distributed and the GlnR-mediated global regulatory system is highly conserved in actinomycetes (Amon et al., 2008; Tiffert et al., 2008). The GlnR binding *cis*-element was first defined by Tiffert et al. (2008), which was comprised of two 22-bp GlnR boxes, each consisting of one “*a* site” of “gTnAc” and one *b* site of “GaAAc” separated by six nucleotides (“5-nt *a* site-n6–5-nt *b* site-n6”). However, with more and more GlnR targets characterized, more complicated GlnR-binding sequences are found, which may consist of either triple 22-bp GlnR boxes in the *amtB* promoter (Wang et al., 2012) or merely two *a* sites, separated by variable length in the promoters of *nasA* (Wang and Zhao, 2009) and *SCO5163* (Lewis et al., 2011) in *S. coelicolor*. Although information-based models were later established to describe GlnR box as consisting of two 11-nt direct repeats (Sola-Landa et al., 2013), the new models were still unable to explain the GlnR-binding *cis*-elements observed in *nasA* and *SCO5163* promoters in *S. coelicolor*. Besides, in *Streptomyces venezuelae*, the most common GlnR binding motif is composed of double *a* sites (GTnAC-n6-GTnAC), and in some cases, no conserved GlnR binding consensus sequences can be identified (Pullan et al., 2011). For the *nas* operon in the *Amycolatopsis mediterranei*, three GlnR binding sites (*a1-b1-b2* sites) were required for GlnR-mediated activation of its transcription, whereas the *a2* site was non-essential (Wang et al., 2013). Therefore, the GlnR binding *cis*-elements are complicated, and more work needs to be done for better understanding of the molecular mechanism of GlnR-mediated transcriptional regulation, especially for targets with non-standard GlnR binding *cis*-elements.

In *S. coelicolor*, GlnR has been shown to directly regulate the transcription of the *amtB-glnK-glnD* operon (*amtB* operon) through binding to its promoter region (Wang et al., 2012; Sola-Landa et al., 2013). Besides of the two typical 22-bp GlnR boxes, Wang et al. (2012) ever characterized an extra GlnR box consisting of *a3*-*b3* sites. However, the biological function and the advantage of the three GlnR boxes in activation of *amtB* transcription remained unclear. We here systematically studied the GlnR binding activities against the three GlnR boxes in *amtB* promoter and investigated the roles of the three GlnR boxes in the transcriptional regulation of *amtB*.

## Materials and Methods

### Bacterial Strains, Media, and Primers

*Escherichia coli* strains were cultured at 37°C with Luria-Bertani medium. The wild-type *S. coelicolor* M145 and its derivatives were cultivated at 30°C on mannitol soya agar for spore suspension preparations (Hobbs et al., 1989). For hygromycin reporter assay and qPCR assay, *S. coelicolor* M145 and its derivatives were grown in either rich S medium (Okanishi et al., 1974) or nitrogen-limited N-Evans medium (Fink et al., 2002) with 5 mM sodium nitrate (poor nitrogen source) supplemented as the sole nitrogen sources. When needed, apramycin (50 μg/ml), kanamycin (50 μg/ml), chloramphenicol (34 μg/ml), ampicillin (100 μg/ml), nalidixic acid (50 μg/ml), and hygromycin B (50 μg/ml) were supplemented into the media. All primers used in this study were listed in **Supplementary Table S1**.

### Electrophoretic Mobility Shift Assay

Recombinant *S. coelicolor* GlnR was expressed in *E. coli* BL21(DE3) and purification process was the same as previously described (Shao et al., 2015). To prepare 6-carboxyfluorescein (FAM)-labeled probes for electrophoretic mobility shift assay (EMSA), primer FAM-M13F-47 was annealed with synthesized *amtB* oligos, followed by strand extension with Taq DNA polymerase (Tolo Biotech). Because the PCR products were so short that were difficult to be purified, we added the probes in the binding reactions based on the concentration of synthesized oligos used in PCR which were quantified with NanoDrop 2000c. The binding of His-tagged GlnR to FAM-labeled probes was performed at room temperature in a total volume of 20 μl containing 50 mM Tris–HCl (pH 8.0), 100 mM KCl, 2.5 mM MgCl_2_, 1 mM dithiothreitol, and 10% glycerol. In all reactions, sheared salmon sperm DNA was added to a final concentration of 100 ng/μl to prevent non-specific binding of the probes by GlnR. After 20-min incubation, probes were separated by a 5% non-denaturing polyacrylamide gel buffered with 1× Tris-acetate-EDTA. Gels were scanned with ImageQuant LAS 4000 mini (GE Healthcare).

### The Hygromycin Reporter System

The schematic chart of plasmid construction was shown in **Supplementary Figure S1**. Specifically, the coding sequence of hygromycin B resistance gene *hyg* was amplified from pML814 (Amon et al., 2008) with primers Rshyg-4 and Rshyg-7, which was then digested with *Xba*I and *Spe*I before being introduced into the *Xba*I site of pSET152 (Bierman et al., 1992). The inserted *hyg* was checked *via Spe*I digestion, and only plasmids with *hyg* gene inserted in an inverted direction with the *int* gene were selected and verified by DNA sequencing with the correct plasmid named as pCMG201. With two pairs of primers, 201T1-F/201T1-A and 201ter-F/201ter-A, two terminators of *rrb*-T1 and T4-g32 were then added to the 5′ and 3′ termini of *hyg*, respectively, employing the site-directed mutation method described before (Wang et al., 2012). The obtained plasmid was designated as pCMG202, which can be used as a reporter system in φC31 *attB* containing strains, e.g., *S. coelicolor*.

The 503-bp promoter region of the *amtB* operon was amplified with primers of amtB-*Eco*RV-F and amtB-*Xba*I-R, and the product was then cloned to the *Hin*cII site of pMZ (Wang et al., 2013), producing pMZ-*amtBp*. The inserted fragment was sequenced, and the direction of the insertion was verified to ensure that the promoter direction was from T7 to T3. Site-directed mutations of the regions in the *amtB* promoter were achieved by PCR using paired primers that contained mutated nucleotides at the 5′ ends, generating pMZ-*amtBp*-a3m, pMZ-*amtBp*-b3m, pMZ-*amtBp*-a1m, pMZ-*amtBp*-b1m, pMZ-*amtBp*-a2m, pMZ-*amtBp*-b2m, pMZ-*amtBp*-m1, pMZ-*amtBp*-m2, and pMZ-*amtBp*-m3. The mutagenesis procedures were the same as previously described (Wang et al., 2012). Both wild-type and mutated fragments were digested with *Eco*RV and *Xba*I and then introduced into the same sites in pCMG202, obtaining a series of pCMG202-derived plasmids. The inserted fragments were sequenced, and all of the constructed plasmids were introduced into *S. coelicolor* M145 by conjugation from *E. coli* ET12567/pUZ8002 (Gust et al., 2003), and confirmed by PCR analysis followed by Sanger sequencing. The conjugated strains were designated as SCamtBp, SCamtBp-a3m, SCamtBp-b3m, SCamtBp-a1m, SCamtBp-b1m, SCamtBp-a2m, SCamtBp-b2m, SCamtBp-m1, SCamtBp-m2, and SCamtBp-m3.

*Streptomyces* spores were quantitated through serial dilution and colony count before being plated onto plates. The same amount of spores were then serially diluted and dotted onto the nitrogen-limited N-Evans medium with the 5 mM nitrate. Either apramycin (50 μg/ml) or hygromycin B (50 μg/ml) was supplemented, which was indicated in the figure legends.

### DNase I Footprinting Assay

To analyze the GlnR-protected regions in both wild-type and mutated promoter regions of *S. coelicolor amtB* operon, DNase I footprinting assay was employed, which was performed by Tolo Biotech. Besides of pMZ-*amtBp*-a2m, *a2* site was also mutated to other sequences, including poly As, poly Ts and poly Cs, respectively. Briefly, plasmid pMZ-amtBP was taken as the template for PCR amplification, using paired primers of amtB-a2m-5a-F, amtB-a2m-5t-F, amtB-a2m-5c-F, and amtB-a2m-R, respectively. The PCR amplicons were then purified and self-ligated at the presence of T4PNK (NEB) and T4 DNA ligase (Tolo Biotech). After that, the ligation products were transformed into *E. coli* DH10B, and the transformants were verified by DNA sequencing with correct plasmids named as pMZ-amtBP-5a, pMZ-amtBP-5t, and pMZ-amtBP-5c, respectively.

To prepare DNA probes for DNase I footprinting assay, both wild-type and mutated promoter regions of *amtB* was first PCR amplified with the primers of SCamtBFP(M13F) and amtB-*Xba*I-R, and the amplicon was then used as the template for further preparation of FAM-labeled probes with primer pairs of amtB-*Xba*I-R and M13F-FAM. PCR products were purified with Wizard SV gel and the PCR Clean-Up system (Promega) and quantified with NanoDrop 2000c. The probe (300 ng) was incubated with different amounts of recombinant GlnR protein in a total volume of 40 μl in the buffer same as described in EMSA. Further random digestion by DNase I, electrophoresis with ABI3130, preparation of the sequencing ladder, and data analysis were carried out following the same procedure as previously described (Wang et al., 2012).

### Construction of the *S. coelicolor* Mutant SCamtBp-m3-gn

Mutation of GlnR boxes in the genome of *S. coelicolor* M145 was achieved by the way of one-step high-efficiency CRISPR/Cas9-mediated genome editing system (Huang et al., 2015). In brief, the sgRNA sequence was amplified with Overlap-m3-sgRNA and Overlap-gTEMDN, employing pKCcas9 as the template (Huang et al., 2015). Then, two pairs of primers Overlap-m3-up-F/Overlap-m3-up-R and Overlap-m3-down-F/Overlap-m3-down-R were used to amplify the upstream and downstream homologous arms, using M145 genomic DNA as the template. The three fragments were purified and used as templates for subsequent overlapping PCR amplification, using primers of Overlap-m3-sgRNA and Overlap-m3-down-R. The amplicon was then digested with *Spe*I and *Hin*dIII, before being inserted into the same sites of pKCcas9. The obtained plasmid was first verified by DNA sequencing and was then conjugated into *S. coelicolor* M145 from *E. coli* ET12567/pUZ8002. The conjugants were verified by PCR analysis and subsequent DNA sequencing. Correct mutant, where *a1-b1* sites were deleted, was named as SCamtBp-m3-gn.

### Real-Time Reverse Transcription PCR

*Streptomyces coelicolor* strains were first cultured at 30°C shaker in liquid S medium for 18 h till the cells entered middle-exponential phase. Then, cells were collected and washed twice with Evans minimal medium without any nitrogen sources, followed by being inoculated into fresh Evans medium with 5 mM sodium nitrate as the sole nitrogen source. Cells were collected by centrifugation at 4°C at the time points of 0 and 120 min, respectively, and then quickly stored in liquid nitrogen.

Total RNA was extracted by using TRIzol (Thermo Fisher Scientific) and SV Total RNA Isolation System (Promega). DNA contamination was removed by digestion with recombinant DNase I (TaKaRa). One microgram of total RNA was used for cDNA synthesis with 6-bp random primers using the PrimeScript^TM^ 1st Strand cDNA Synthesis Kit (TaKaRa) in a 20 μl reaction system, following the manufacturer’s protocol. Real-time PCR assay was carried out with SYBR^®^ Premix Ex Taq^TM^ II (TaKaRa) according to the manufacturer’s instructions. At least three independent samples were tested, and the fold change of relative transcript levels of gene *amtB* and *glnA* in response to deficient nitrogen source in 120 to 0 min between the WT and SCamtB-m3-gn strains was expressed as mean ± standard deviation (SD), employing the transcription of *hrdB* as an internal control. *t*-Test was performed to test the difference.

## Results

### GlnR Shows Different Binding Affinities against the Three GlnR Boxes in *amtB* Promoter

**FIGURE 1:**
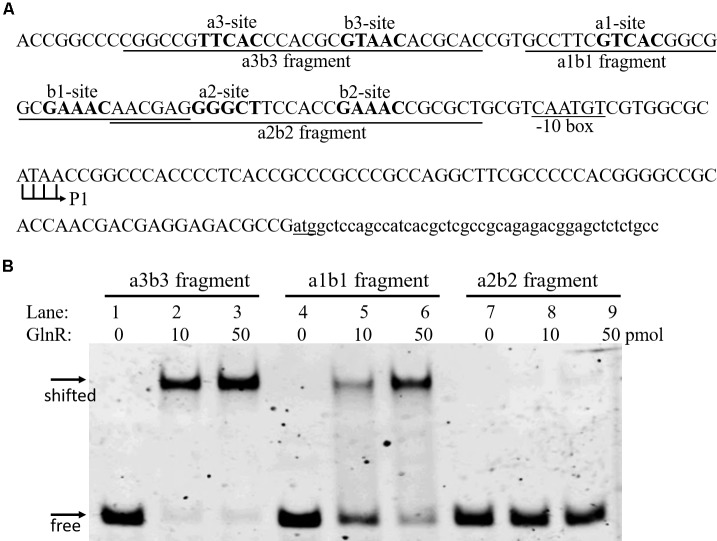
Characterization of the GlnR binding affinity against each GlnR box in *amtB* promoter. **(A)** DNA sequences in *amtB* promoter region. Both *a* sites and *b* sites were shown in bold. Three fragments for EMSA were labeled and indicated by horizontal solid lines. The translation start site (atg) was underlined, and both the transcription initiation site (P1) and the predicted –10 box were labeled. **(B)** EMSA with GlnR and probes shown in **(A)**. Three different amounts of GlnR were employed, and sheared salmon sperm DNA was added to prevent non-specific binding of GlnR to the probes.

Three GlnR boxes were found in the promoter of *amtB* operon, each consisting of one *a* site and one *b* site (*a3-b3*, *a1-b1*, and *a2-b2*; **Figure 1A**). This three-GlnR-box model was conserved in *amtB* promoter among *Streptomyces*, which was distinct from most other GlnR targets, where usually two GlnR boxes were identified (**Supplementary Figure S2**). To reveal the role of each GlnR box in GlnR binding of *amtB* promoter, we measured the GlnR binding affinity against each box. Three synthesized DNA fragments, each of which contained an individual GlnR box (i.e., *a3-b3*, *a1-b1*, and *a2-b2*), were employed for EMSA with GlnR. The results showed that GlnR was able to bind *a3-b3* and *a1-b1*, and the binding affinity against *a3-b3* was higher. However, *a2-b2* alone could hardly be bound by GlnR (**Figure 1B**).

To reveal the cause of the low binding affinity between GlnR and *a2-b2* sites, we compared the upstream regions of the *amtB* operon among *Streptomyces* through sequence alignment analysis (**Supplementary Figure S2A**), and found that the *a2* site was not a typical GlnR binding site, which also indicated that the six GlnR binding sites might show different binding affinities with GlnR.

### *a2* Is Dispensable for GlnR-Mediated Activation of *amtB* Transcription *in Vivo* and GlnR Binding *in Vitro*

**FIGURE 2:**
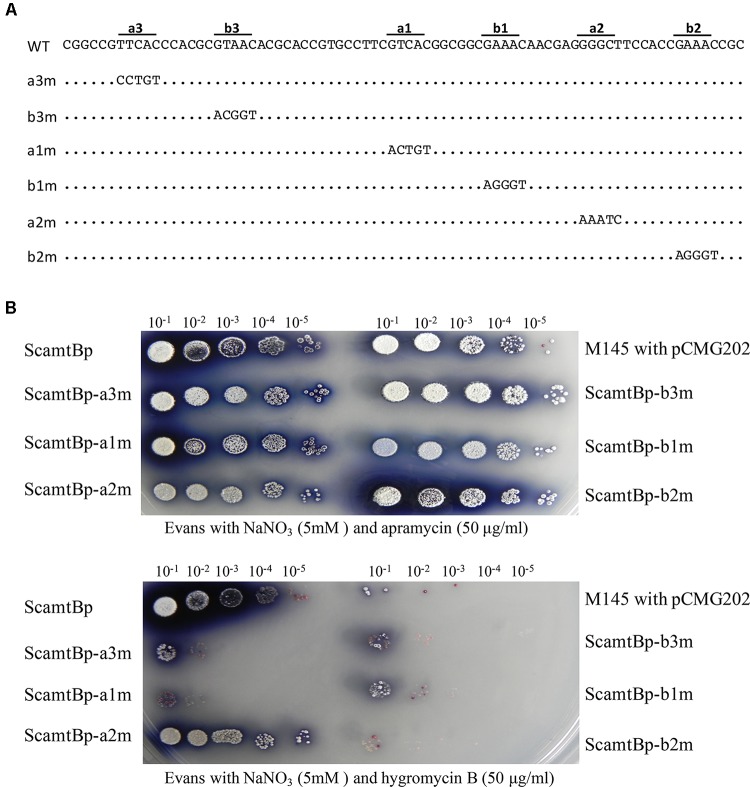
Analysis of the *in vivo* transcriptional activities of both the wild-type and mutated *amtB* promoters with the hygromycin reporter system. M145 derivatives were grown on N-Evans medium with 5 mM sodium nitrate as the sole nitrogen source, and either **(A)** 50 μg/ml apramycin or **(B)** 50 μg/ml hygromycin B was supplemented. The *aac(3)IV* gene on the reporter plasmid enabled the conjugants to grow under the apramycin conditions, which was employed as a positive control. The ability to grow on hygromycin B medium reflected the expression level of the *hyg* gene as well as the transcriptional activities of the tested promoters. Quantification and gradient dilution of spores were described in Section “Materials and Methods.” Photos were taken after 5 days’ incubation at 30°C.

To further characterize the six GlnR binding sites, base transition mutation of each site was individually performed, generating six mutated promoters (**Figure 2A**). We then developed a reporter system to test the *in vivo* functions of these six GlnR binding sites, employing hygromycin B resistance gene (*hyg*), which encodes a phosphotransferase and inactivates hygromycin through phosphorylating a hydroxyl group, as the reporter gene. Mutated *amtB* promoters together with the wild-type promoter were inserted into the reporter system, and the reporter plasmids were then conjugated into *S. coelicolor* to test the bacterial resistance to hygromycin B, which could reflect the promoter activities. The results clearly showed that only *a2* mutation had no effect on bacterial growth, while the other five mutations severely impeded bacterial growth in medium with hygromycin (**Figure 2B**), indicating that *a2* site was dispensable.

**FIGURE 3:**
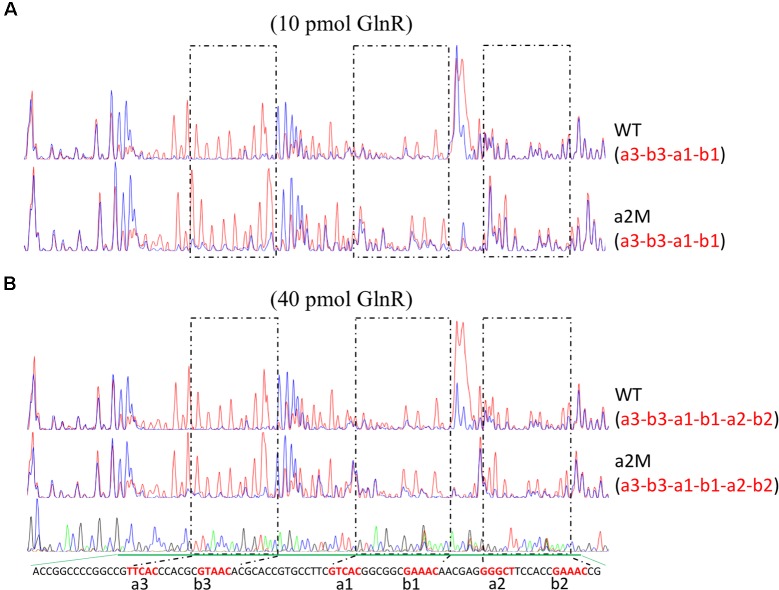
Characterization of the GlnR binding sites in *amtB* promoter through *in vitro* DNase I footprinting assay. DNase I footprinting analyses of the GlnR-protected DNA sequences in both the wild-type and *a2*-mutated *amtB* promoter regions. Different amounts of GlnR protein were used, including both 10 pmol **(A)** and 40 pmol **(B)**. The GlnR binding boxes were marked with dashed boxes and labeled in red in the sequencing panel below each figure. For each assay, the GlnR-protected sites were indicated in the bracket below the name of the probe. Red lines: assays without GlnR; blue lines: assays with GlnR.

Furthermore, we also employed the DNase I footprinting assay to accurately determine the binding affinity of GlnR to the wild-type and *a2*-mutated promoters, with two different concentrations of GlnR assayed (**Figure 3**). For wild-type *amtB* promoter, *a3-b3* and *a1-b1* could be protected by GlnR under a low concentration (0.2 pmol/μl), while the protection of *a2-b2* required an increased concentration of GlnR (0.8 pmol/μl). This result was consistent with those of EMSA, where GlnR bound *a2-b2* with the least binding affinity (**Figure 1B**). When *a2* was mutated (**Figure 3** and **Supplementary Figure S3**), the GlnR-protected region was the same as that in the wild-type promoter, which was highly consistent with the *in vivo* reporter assay, indicating that *a2* was dispensable for GlnR binding of *amtB* promoter *in vitro*.

### The GlnR Binding Affinity Is Important for GlnR-Mediated Activation of *amtB* Transcription

**FIGURE 4:**
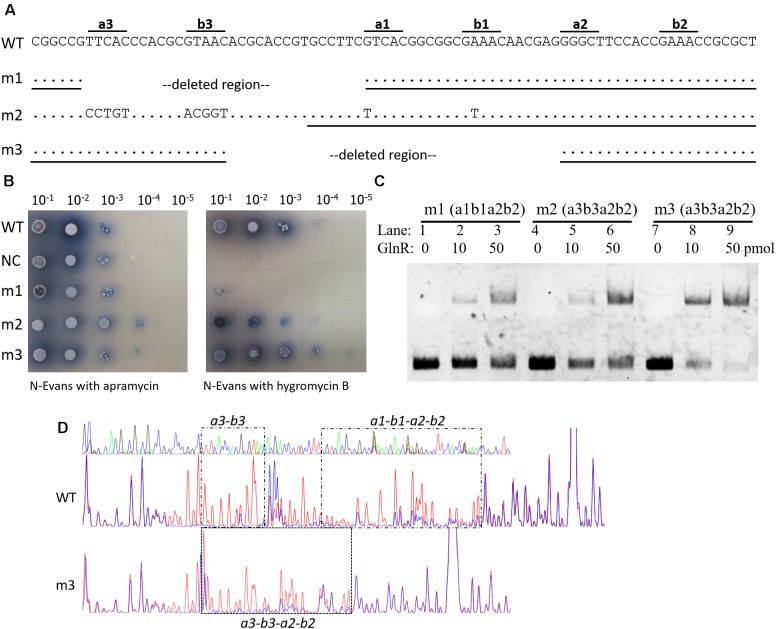
Engineering of an *amtB* promoter with two GlnR boxes. **(A)** Illustration of the construction of three two-GlnR-box *amtB* promoters (i.e., m1 to m3), and deleted regions and mutated sequences were labeled in each mutated promoter. DNA sequences used for EMSA in **(C)** were indicated with black horizontal lines. **(B)** Analysis of the promoter activities of the three mutated *amtB* promoters. The wild-type promoter (WT) was employed as a positive control, while the reporter plasmid with no promoter (NC) was employed as a negative control. Serially diluted spores were dotted on N-Evans medium with 5 mM sodium nitrate as the sole nitrogen source, and either 50 μg/ml apramycin or 50 μg/ml hygromycin B was supplemented. Photos were taken after 5 days’ incubation at 30°C. **(C)** EMSA analysis of the GlnR binding affinity against each mutated promoter. Different amounts of GlnR protein were used, and sheared salmon sperm DNA was added in each reaction system to prevent non-specific binding. **(D)** DNase I footprinting analysis of the GlnR-protected DNA sequences in the wild-type and m3 promoter regions of *amtB*. The GlnR binding sites were marked with black dashed boxes and labeled either above or below the boxes.

Typical GlnR target promoters contain two GlnR boxes (i.e., the two-GlnR-box motif), such as *glnA*, *glnII*, and *nirB*, but *amtB* promoter has an extra GlnR box (**Supplementary Figure S2B**). To further study the biological roles of triple GlnR boxes in *amtB* promoter, we mutated the *amtB* promoter and reconstructed it as a typical GlnR target promoter, which had only two GlnR boxes (**Figure 4A**). Then, the transcriptional activities of the mutated promoters were tested by the *hyg* reporter system (**Figure 4B**).

We first deleted *a3-b3* to generate the *amtB* promoter mutant 1 (m1), and found the reporter gene failed to be activated in m1. This finding was consistent with the results we observed before (Wang et al., 2012), where mutation of *a3-b3* in *amtB* promoter resulted in the failure in GlnR-mediated transcriptional activation of *amtB*. When *a3-b3* was mutated and meanwhile *a1-b1* was mutated to *a3-b3*, the obtained promoter was designated as *amtB* promoter mutant 2 (m2), which had only *a3-b3* and *a2-b2*. Interestingly, m2 could be moderately activated by GlnR. However, when we deleted a region containing *a1-b1* sites in the wild-type promoter, which exactly moved *a3-b3* to the position of *a1-b1*, the generated *amtB* promoter mutant 3 (m3) could also be activated by GlnR to an extent comparable to the wild-type. Notably, in both m2 and m3 promoters, the distance between *b3* site and *a2* site was 6 nt, a previously well-defined distance between GlnR binding sites (Tiffert et al., 2008; Wang et al., 2015), making *a3-b3–a2-b2* a typical GlnR box.

We further confirmed the GlnR binding affinity against each mutated promoter with EMSA, and found GlnR had the highest binding affinity for m3 while the lowest for m1 (**Figure 4C**), which was in high accordance with the *in vivo* promoter activities (**Figure 4B**), suggesting the GlnR binding affinity to the target promoter was the key requirement for GlnR-mediated transcriptional activation. Moreover, with DNase I footprinting assay, we found the GlnR-protected region in m3 fully covered *a3-b3*–*a2-b2* sites, which was correspondent with our expectation (**Figure 4D**).

### Three GlnR Boxes Assist GlnR in More Promptly Activating *amtB* Transcription

Based on the results of the above reporter assay, m3 could be normally activated by GlnR to an extent comparable to the wild-type. However, its transcriptional activity was not quantitatively determined. To precisely measure the m3 transcriptional activity and compare the differences between m3 and the wild-type, we *in situ* constructed the m3 mutation in the genome of *S. coelicolor* M145, generating strain SCamtB-m3-gn (m3 mutant in genome). Then, we measured the *amtB* transcription in both m3 mutant and M145, responding to nitrogen limitation.

**FIGURE 5:**
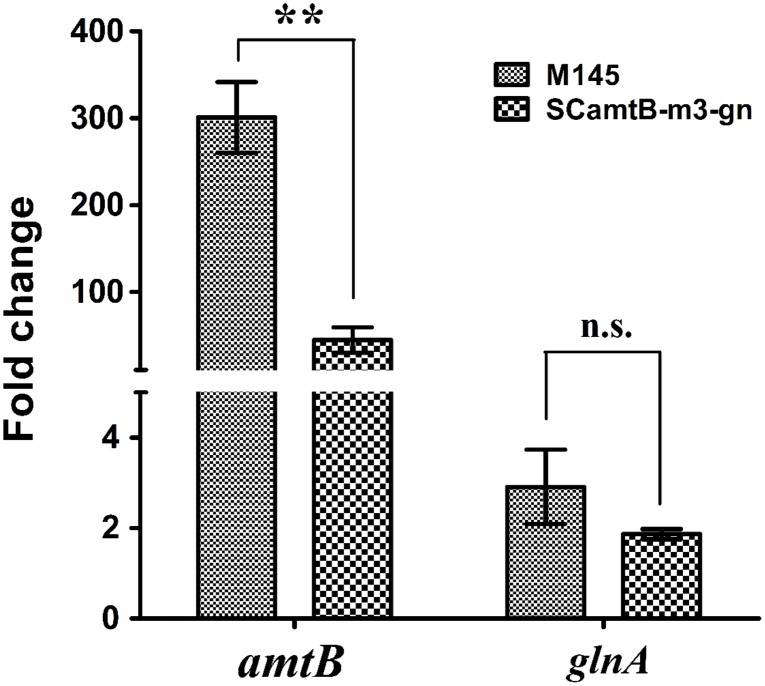
Time-course analysis of the relative transcriptional levels of *amtB* in both wild-type and SCamtB-m3-gn (m3 mutant). From time 0 min, cells were transferred to N-Evans medium with 5 mM sodium nitrate as the sole nitrogen source, and cells were harvested for transcriptional analysis at the time 120 min. As a representative of two-GlnR-box promoters, *glnA* was employed as a control. The transcriptional level of *amtB* and *glnA* at time 0 min was normalized to 1.0, and SD as well as error bars were calculated with data from three independent experiments. ^∗∗^*P* < 0.01; n.s., no significant; *t*-test.

Strains were first cultured in rich S medium and then transferred to minimal Evans medium with 5 mM nitrate, mimicking nitrogen-limited conditions. With the employment of real-time reverse transcription PCR, we found that although *amtB* transcription rapidly increased in both strains after the addition of nitrate, the increase at the time of 60 min relative to 0 min was much higher in the wild-type than in m3 mutant (i.e., 300 folds in M145 *versus* 45 folds in m3 mutant; **Figure 5**). As a control, the transcriptional level of the unmodified *glnA* was almost the same in both strains. Notably, as a representative of the typical GlnR targets with a two-GlnR-box motif, *glnA* was moderately regulated (i.e., twofold of activation; **Figure 5**). Taken together, the above results proved that three GlnR boxes enabled more prompt and greater activation of *amtB* transcription in response to nitrogen limitation.

## Discussion

A classic GlnR binding *cis*-element is comprised of two GlnR boxes, each consisting of one “*a* site” and one “*b* site.” However, with the characterization of more and more GlnR targets, the GlnR binding features are now well-known to be complicated. Among GlnR targets, the *amtB* promoter contains three pairs of GlnR boxes, i.e., six GlnR binding sites, which is so far the largest number of GlnR sites found within one promoter. In this study, we demonstrated that *a2* site was not essential for GlnR-mediated binding of *amtB* promoter *in vitro* and transcriptional activation of *amtB in vivo*. Although probe *a2-b2* was almost the same as probe *a1-b1*, it could not be normally bound by GlnR, which was distinct from *a1-b1*. Therefore, we concluded that the non-conservative *a2* site led to the failure in binding of *a2-b2* alone by GlnR. As *b2* site was demonstrated as an essential site, the above results were therefore not contradictory with our previous findings that the GlnR box2 comprised of *a2-b2* sites was essential for *amtB* transcription, where mutation of both *a2* and *b2* sites led to inactivation of *amtB* promoter (Wang et al., 2012). Moreover, in our previous study, the *a2* site in the promoter of *nas* operon in *A. mediterranei* U32 was also proved to be non-essential for both GlnR binding *in vitro* and GlnR-mediated transcriptional regulation *in vivo* (Wang et al., 2013).

In *amtB* promoter, *a2* site locates just at the position of a classical -35 element (**Figure 1**). Based on the classic model of transcriptional regulation, occupation of *a2* by GlnR may prevent the σ subunit of the RNA polymerase (RNAP) from recognizing the -35 element. Therefore, GlnR may interact with the σ subunit to recruit RNAP to activate the *amtB* transcription (Browning and Busby, 2004). However, there exist 67 σ factors in *S. coelicolor*, and it is still unclear which σ factor is responsible for transcriptional regulation of nitrogen-related genes. Therefore, further characterization of the σ factor that participates in the transcription of *amtB* operon may facilitate better understanding of the GlnR-mediated transcriptional regulation of *amtB* transcription.

Besides, as *a3-b3* sites are competitively bound by GlnR and PhoP (a global regulator for phosphate metabolism) in *S. coelicolor*, *amtB* operon is therefore regulated by both regulators responding to both signals of nitrogen and phosphate availabilities (Wang et al., 2012; Sola-Landa et al., 2013). GlnR functions as a transcriptional activator (e.g., for most GlnR targets) or repressor (e.g., for *gdhA*), and regulates the transcription of GlnR regulon under nitrogen-limited conditions (Fink et al., 2002; Tiffert et al., 2008, 2011). While PhoP governs the phosphate metabolisms and regulates the transcription of Pho regulon under phosphate limitation (Sola-Landa et al., 2005, 2013; Apel et al., 2007; Rodriguez-Garcia et al., 2009; Martin et al., 2017). Because *a3-b3* sites are essential for the activation of *amtB* transcription (Wang et al., 2012), the competitive binding of *a3-b3* between GlnR and PhoP determines the transcriptional level of *amtB* operon, and only when GlnR occupies *a3-b3*, the transcription of *amtB* is activated.

Although the influence of phosphate supply on *amtB* transcriptional regulation was not tested in this study, the results here clearly indicated the cross-regulation between phosphate and nitrogen metabolisms in *S. coelicolor*. For example, when phosphate is in excess but nitrogen is limited, GlnR activates *amtB* transcription to increase the intracellular nitrogen supply, helping bacteria cope with the stress of nitrogen limitation. Otherwise, when *S. coelicolor* is cultured in phosphate-limited medium, PhoP may compete against GlnR to occupy *a3-b3*, which will repress the expression of *amtB* operon to slow down the nitrogen metabolisms, obtaining intracellular nitrogen/phosphate balance.

Taken together, the existence of the extra GlnR box (*a3-b3*) in *amtB* promoter not only builds a perfect connection between nitrogen and phosphate metabolisms but also assists in more prompt activation of *amtB* transcription in response to nitrogen availability.

## Author Contributions

ZL and XL performed most of the experiments. JzW performed the DNase I footprinting assay. YW constructed the hygromycin reporter system. GsZ helped in the construction of mutant SCamtBp-m3-gn. ZL drafted the manuscript. YL, GpZ, and JW analyzed the data and revised the manuscript. JW supervised the study.

## Conflict of Interest Statement

The authors declare that the research was conducted in the absence of any commercial or financial relationships that could be construed as a potential conflict of interest.

## Supplementary Material

FIGURE S1 Schematic chart for construction of pCMG202. The detailed procedure could be found in the Section “Materials and Methods.”

FIGURE S2 Analysis of the GlnR binding sites in the promoter regions of GlnR targets. **(A)** Comparison of the upstream regions of the *amtB* operon among *Streptomyces*. The conserved *a3*, *b3*, *a1*, *b1*, and *b2* sites were indicated by black boxes, while the *a2* site was not a typical GlnR binding site and was indicated by a gray box. Symbols used: SCO, *Streptomyces coelicolor*; SAV, *Streptomyces avermitilis*; SCAB, *Streptomyces scabies*; SCLAV, *Streptomyces clavuligerus*; SGR, *Streptomyces griseus*; SLI, *Streptomyces lividans*; STRS, *Streptomyces* spp.; SVEN, *Streptomyces venezuelae*. **(B)** Comparison of the upstream regions of the GlnR target genes in *S. coelicolor*. The conserved *a1-b1* and *b2* sites were indicated by black boxes, while the *a3-b3* and *a2* sites were not conserved and were indicated by gray boxes.

FIGURE S3 Characterization of the GlnR-protected regions in both wild-type and *a2*-mutated *amtB* promoters. The *a2* site was mutated to “AAAAA,” “CCCCC,” or “TTTTT,” respectively. For DNase I footprinting assays, mutated promoters were incubated with GlnR protein, employing the wild-type promoter as a positive control. As *a2* site (“GGGCT”) contained several Gs, the site was thus not mutated to poly Gs. Assays without GlnR were shown in red lines, and blue lines indicated assays with 40 pmol GlnR.

TABLE S1 Primers used in this study.
