# Supplementary figures and images for: Insight into the Molecular Mechanism of the Transcriptional Regulation of amtB Operon in Streptomyces coelicolor
Source: Front Microbiol. 2018 Feb 20;9:264. doi: 10.3389/fmicb.2018.00264 (PMC5826061; doi:10.3389/fmicb.2018.00264)

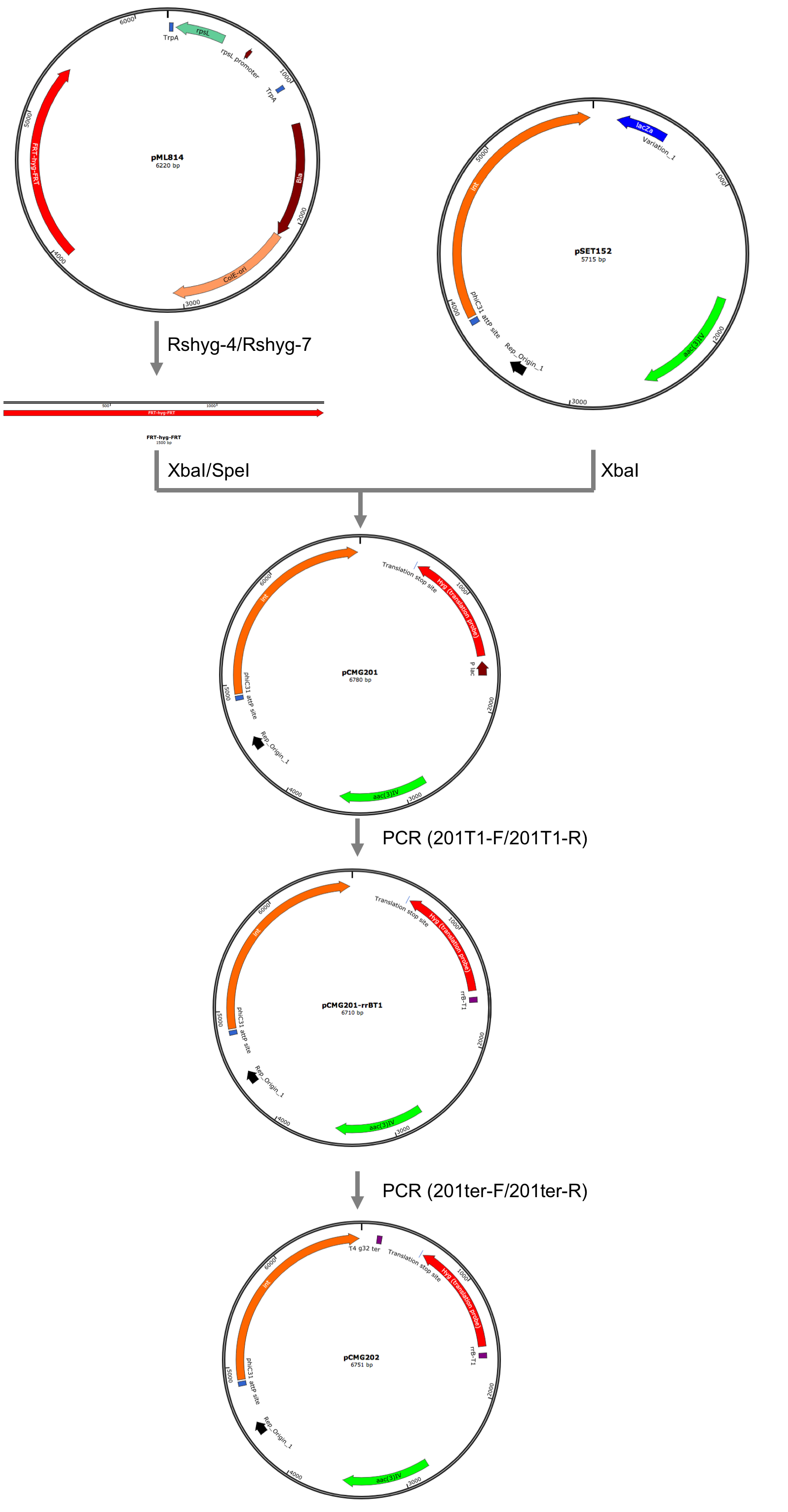

Supplement: FIGURE S1 — Schematic chart for construction of pCMG202. The detailed procedure could be found in the Section “Materials and Methods.” [file Image_1.TIF]

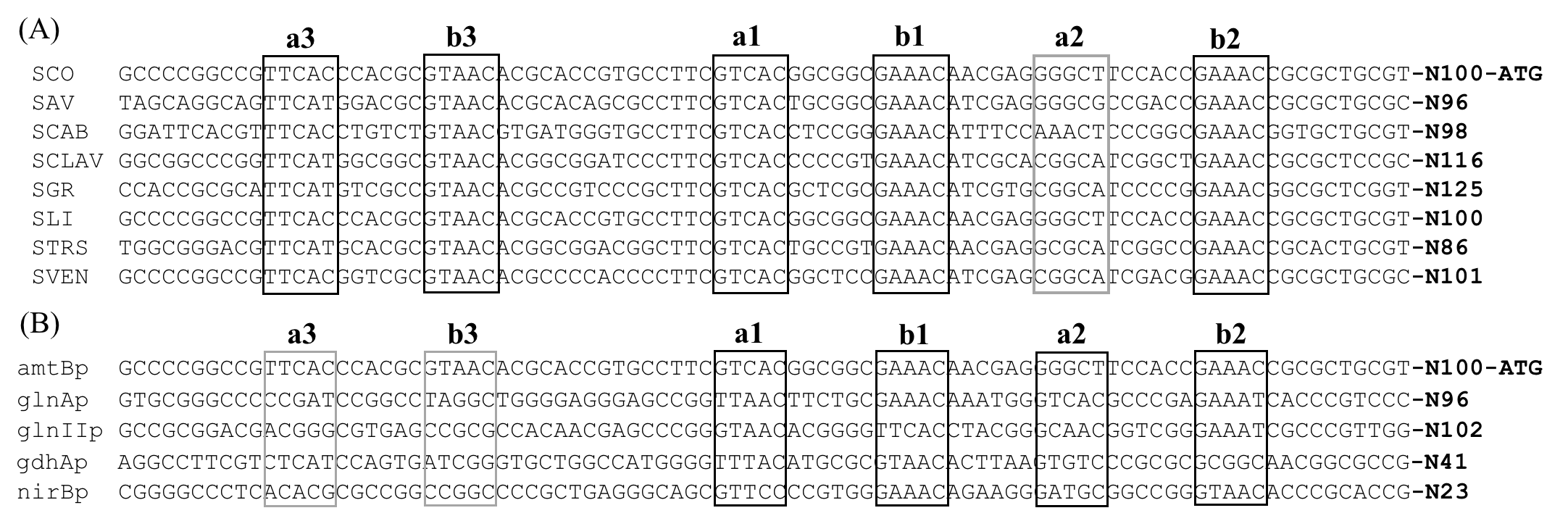

Supplement: FIGURE S2 — Analysis of the GlnR binding sites in the promoter regions of GlnR targets. (A) Comparison of the upstream regions of the amtB operon among Streptomyces. The conserved a3, b3, a1, b1, and b2 sites were indicated by black boxes, while the a2 site was not a typical GlnR binding site and was indicated by a gray box. Symbols used: SCO, Streptomyces coelicolor; SAV, Streptomyces avermitilis; SCAB, Streptomyces scabies; SCLAV, Streptomyces clavuligerus; SGR, Streptomyces griseus; SLI, Streptomyces lividans; STRS, Streptomyces spp.; SVEN, Streptomyces venezuelae. (B) Comparison of the upstream regions of the GlnR target genes in S. coelicolor. The conserved a1-b1 and b2 sites were indicated by black boxes, while the a3-b3 and a2 sites were not conserved and were indicated by gray boxes. [file Image_2.TIF]

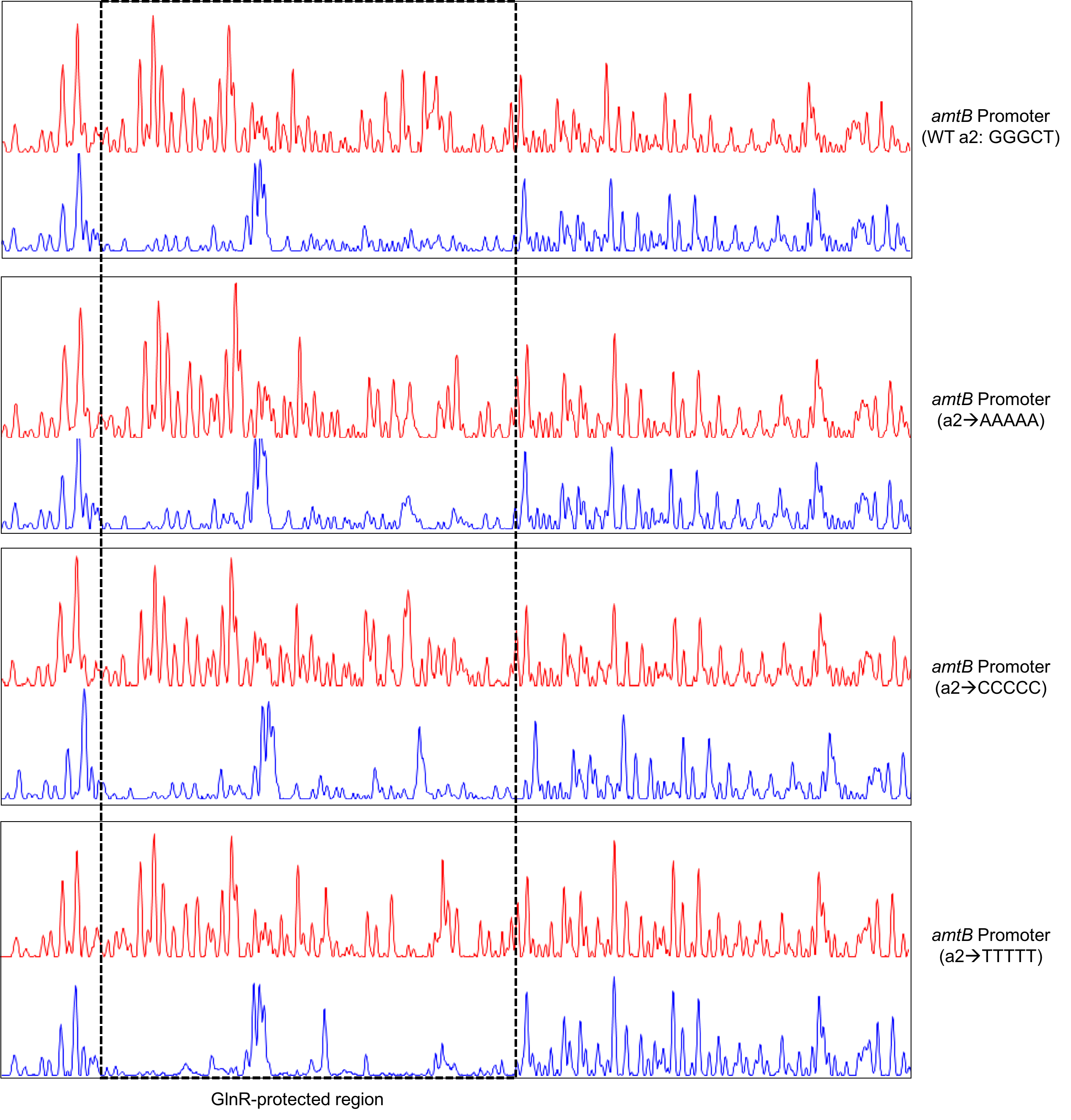

Supplement: FIGURE S3 — Characterization of the GlnR-protected regions in both wild-type and a2-mutated amtB promoters. The a2 site was mutated to “AAAAA,” “CCCCC,” or “TTTTT,” respectively. For DNase I footprinting assays, mutated promoters were incubated with GlnR protein, employing the wild-type promoter as a positive control. As a2 site (“GGGCT”) contained several Gs, the site was thus not mutated to poly Gs. Assays without GlnR were shown in red lines, and blue lines indicated assays with 40 pmol GlnR. [file Image_3.TIF]
