# Supplementary material for: Insight into the Molecular Mechanism of the Transcriptional Regulation of amtB Operon in Streptomyces coelicolor
Source: Front Microbiol. 2018 Feb 20;9:264. doi: 10.3389/fmicb.2018.00264 (PMC5826061; doi:10.3389/fmicb.2018.00264)
Supplement: TABLE S1 — Primers used in this study. [file Table_1.DOCX]

**Table S1. Primers used in this study**

| **Names** | **Sequences (5’-3’)** | | | |  |  |  |
| --- | --- | --- | --- | --- | --- | --- | --- |
| **Oligonucleotides for ds-DNA annealing for EMSA (with M13-47 reverse complementary sequences underlined)** | | | | |  |  |  |
| amtBP-a3b3 | CGGCCGTTCACCCACGCGTAACACGCACGTCGTGACTGGGAAAACCCTGGCG | | | |  |  |  |
| amtBP-a1b1 | GCCTTCGTCACGGCGGCGAAACAACGAGGTCGTGACTGGGAAAACCCTGGCG | | | |  |  |  |
| amtBP-a2b2 | AACGAGGGGCTTCCACCGAAACCGCGCTGTCGTGACTGGGAAAACCCTGGCG | | | |  |  |  |
| amtBP-m1 | GCCTTCGTCACGGCGGCGAAACAACGAGGGGCTTCCACCGAAACCGCGCTGTCGTGACTGGGAAAACCCTGGCG | | | |  |  |  |
| amtBP-m2 | GCCTTCTTCACGGCGGCGTAACAACGAGGGGCTTCCACCGAAACCGCGCTGTCGTGACTGGGAAAACCCTGGCG | | | |  |  |  |
| amtBP-m3 | CGGCCGTTCACCCACGCGTAACACGCAGGGGCTTCCACCGAAACCGCGCTGTCGTGACTGGGAAAACCCTGGCG | | | |  |  |  |
| M13F-47-FAM | CGCCAGGGTTTTCCCAGTCACGAC (5’ FAM labeled) | | | |  |  |  |
| **PCR amplification of *amtB* promoter region** | | | | |  |  |  |
| *amtB*-EcoRV-F | AAAAAGATATCAACCCGAGGAGAGCACCGTG | | | |  |  |  |
| *amtB*-XbaI-R | AAAAATCTAGACATCGGCGTCTCCTCGTCGTT | | | |  |  |  |
| **Mutation of the promoter region of *amtB* operon** | | | | |  |  |  |
| amtB-a3m-F | | GCCGcctgtCCACGCGTAACACGCACCGT | | | |  |  |
| amtB-a3m-R | | CGGGGCCGGTCGTCGTCGTG | | | |  |  |
| amtB-b3m-F | | GCCGTTCACCCACGCacggtACGCACCGTGCCTTCGTCAC | | | |  |  |
| amtB-a1m-F | | actgtGGCGGCGAAACAACGAGGGG | | | |  |  |
| amtB-a1m-R | | GAAGGCACGGTGCGTGTTAC | | | |  |  |
| amtB-b1m-F | | GTCACGGCGGCagggtAACGAGGGGCTTCCACCGAA | | | |  |  |
| amtB-a2m-F | | aaatcTCCACCGAAACCGCGCTGCG | | | |  |  |
| amtB-a2m-5a-F | | aaaaaTCCACCGAAACCGCGCTGC | | | |  |  |
| amtB-a2m-5t-F | | tttttTCCACCGAAACCGCGCTGC | | | |  |  |
| amtB-a2m-5c-F | | cccccTCCACCGAAACCGCGCTGC | | | |  |  |
| amtB-a2m-R | | CTCGTTGTTTCGCCGCCGTG | | | |  |  |
| amtB-b2m-F | | GGGCTTCCACCagggtCGCGCTGCGTCAATGTCGTG | | | |  |  |
| amtB-m1-F | | GGCCGGTCACGGCGGCGAAACAAC | | | |  |  |
| amtB-m1-R | | GGGGCCGGTCGTCGTC | | | |  |  |
| amtB-m2-F | | GTGCCTTCtTCACGGCGGCGtAACAACGAGGGGCTTCCAC | | | |  |  |
| amtB-m2-R | | GGTGCGTaccgtGCGTGGacaggCGGCCGGGGCCGGTCGTCGTCGT | | | |  |  |
| amtB-m3-F | | GGGGCTTCCACCGAAAC | | | |  |  |
| amtB-m3-R | | TGCGTGTTACGCGTGGGTGAAC | | | |  |  |
| **FAM-labeling of *amtB* promoters for DNase I footprinting assay (with M13F and M13R reverse complementary sequences underlined)** | | | | |  |  |  |
| SCamtBFP(M13F) | | | GTAAAACGACGGCCAGTCCATGCCAGGTCATTCGGAG | | | |  |
| *amtB*-XbaI-R | | | AAAAATCTAGACATCGGCGTCTCCTCGTCGTT | | | |  |
| M13F-FAM | | | GTAAAACGACGGCCAGT (5’-FAM labeled) | | | |  |
| **Construction of SCamtBp-m3-gn** | | | | |  |  |  |
| Overlap-m3-sgRNA | | | | GGACTAGTGTCACGGCGGCGAAACAACGGTTTTAGAGCTAGAAATA | | | |
| Overlap-gTEMDN | | | | CTCAAAAAAAGCACCGACTCGG | | | |
| Overlap-m3-up-F | | | | CCGAGTCGGTGCTTTTTTTGAGTCCAGGAACCCCAGACGGAAACCAC | | | |
| Overlap-m3-up-R | | | | TGCGTGTTACGCGTGGGTGAACGG | | | |
| Overlap-m3-down-F | | | | TCACCCACGCGTAACACGCAGGGGCTTCCACCGAAACCGCGCT | | | |
| Overlap-m3-down-R | | | | CCCAAGCTTTCGTCGTAGCCGAACCTGTACTTCAGG | | | |
| pKCcas9-confirm-F | | | | GCTCAGTCCTAGGTATAAT | | | |
| pKCcas9-confirm-R | | | | CAGTCACGACGTTGTAAAAC | | | |
| Genome-confirm-F | | | | TGCTGGAGTCCACGGCCGTGATGTTC | | | |
| Genome-confirm-R | | | | TGGCGAAGAAGCCGATGAGCAGCGAG | | | |
| Genome-middle-confirm-R | | | | TGCGTGTTACGCGTGGGTGAAC | | | |
| **Real-time RT-PCR** | | | | | | |  |
| hrdB-realtime-F | | | ATTGAGCGGGGAAAGGCTGA | | | |  |
| hrdB-realtime-R | | | GCGCAGTACGTTCTTCCAC | | | |  |
| amtB-realtime-F | | | GCTTCATGCTCATCTGCTCC | | | |  |
| amtB-realtime-R | | | GCATGTTCAGGGTGCTCTTG | | | |  |
| glnA-realtime-F | | | GTCATGCAGCACTTCACGC | | | |  |
| glnA-realtime-R | | | CATGTCCGACTCGTGGATGG | | | |  |
| **Construction of the reporter system** | | | | | | |  |
| Rshyg-4 | | | AATCTAGAGTGACACAAGAATCCCTGTT | | | |  |
| Rshyg-7 | | | TTACTAGTTGGCGGTACTTGGGTCGAT | | | |  |
| 201T1-F | | | ACTGAGCCTTTCGTTTTATACTGCCCGCTTTCCAGTCGGGAAA | | | |  |
| 201T1-R | | | CGGAAGACTGGGCCTTTCGTTTTATGGAAACAGCTATGACATGATTACGAAT | | | |  |
| 201ter-F | | | CCCCTTTTTTATTTTAAAAATTTTTTCAGCTATTTACCCGCAGGACATATC | | | |  |
| 201ter-R | | | ACCTCTAGGGTCCCCCGCCGATGGTTTCTACAAAGATCG | | | |  |
